# Astrocytes: The Unsung Architects of Synaptic Integration and Their Role in Brain Health and Disease

**DOI:** 10.3390/biom15121744

**Published:** 2025-12-16

**Authors:** Rosalie Elvira, Eng King Tan, Zhi Dong Zhou

**Affiliations:** 1 Department of Neurology, National Neuroscience Institute, 11 Jalan Tan Tock Seng, Singapore 308433, Singapore; 2 Department of Neurology, Singapore General Hospital, Outram Road, Singapore 169608, Singapore; 3 Signature Research Program in Neuroscience and Behavioral Disorders, Duke-NUS Medical School, 8 College Road, Singapore 169857, Singapore

**Keywords:** astrocytes, astrocyte leaflets, synaptic integration, neuroinflammation, neurological diseases, therapeutic targeting

## Abstract

Astrocytes, long viewed as passive support cells, are now recognized as dynamic regulators of synaptic function. This mini review summarizes recent findings revealing that astrocyte leaflets, thin peripheral processes of astrocyte, form gap junction interconnected domains containing tiny endoplasmic reticulum saccules. Interestingly, the astrocyte leaflets directly contact and enwrap 90% of neuron synapses in clusters. Furthermore, neuronal circuit activity could trigger local Ca^2+^ dynamics in astrocyte leaflets mediated by inositol 1,4,5-trisphosphate receptor type 1 (IP_3_R1), while leaflet Ca^2+^ signals could integrate neuronal circuits active at different spatiotemporal scales. These findings uncover the novel glial-synapse interactions and highlight the pathophysiological roles of astrocyte leaflets in neuron circuit computation, relevant to human neurological diseases (NDs). In addition, therapeutic opportunities, such as targeting leaflet calcium signaling for neuroprotection, alongside challenges in imaging and modeling are discussed. Future perspectives emphasize advanced tools like optogenetics and artificial intelligence to unravel astrocyte contributions, paving the way for glial-centric interventions in NDs.

## 1. Introduction

The brain’s synaptic networks, comprising trillions of connections, underpin cognition, memory, and behavior. The brain’s synaptic networks, comprising trillions of connections, underpin cognition, memory, and behavior. For decades, neuroscientific research focused predominantly on neurons as the primary architects of these networks. This view was fundamentally reshaped by the tripartite synapse concept, which established the perisynaptic astrocyte as an indispensable third element, dynamically regulating synaptic transmission and plasticity [1]. This paradigm shift was built upon foundational discoveries. Early electron microscopy revealed astrocytic processes ensheathing synapses, suggesting a structural role [2]. Functional studies demonstrated their critical roles in maintaining homeostasis, from clearing neurotransmitters like glutamate to prevent excitotoxicity [3], to buffering ions and modulating extracellular space [4,5]. Crucially, astrocytes were found to be active communicators. They detect synaptic activity via neurotransmitter receptors (e.g., mGluRs) and respond with intracellular calcium elevations, which can trigger the release of gliotransmitters such as ATP and D-serine. The latter acts as a necessary co-agonist for NMDA receptors, making astrocytes essential for the induction of long-term potentiation (LTP), a cellular correlate of learning and memory [5,6,7].

Beyond signaling, astrocytes serve as vital metabolic partners for neurons. They supply energy substrates via the astrocyte-neuron lactate shuttle (ANLS) and provide cholesterol and lipoprotein (e.g., via ApoE) crucial for synaptic membrane synthesis and repair [8,9,10]. Genetic manipulations, such as astrocyte-specific *IP_3_R2* knockout, further highlighted their roles in circuit development, including synapse formation and pruning, for example, complement component (C1q) tagging [11,12,13]. Pathologically, reactive astrocyte states have been implicated in exacerbating toxicity in diseases like Alzheimer’s, through mechanisms such as cytokine release [14] and the potentiation of neurotoxic Aβ production [15].

Despite this comprehensive understanding of astrocyte biology, a critical and persistent gap remained. The precise ultrastructural and functional mechanism by which a single astrocyte integrates information from its thousands of associated synapses to exert coordinated, circuit-level influence was unknown. While the existence of their finest peripheral processes, variously called leaflets or lamellae, has long been recognized from ultrastructural studies [2], they were largely considered passive, uniform extensions. The field lacked the tools to dissect how these nanoscale structures could possibly perform sophisticated integration.

The recent groundbreaking work by Benoit et al. (2025) directly addresses this gap [16]. By applying volumetric high-resolution electron microscopy (vHREM) and advanced functional imaging, their study redefines astrocyte leaflets not as passive sheets, but as organized, gap-junction-linked functional domains containing specialized endoplasmic reticulum (ER) compartments. They identify IP_3_R1, not the somatically dominant IP_3_R2, as the key calcium channel driving local signaling within these leaflets, enabling the integration of synaptic inputs across multiple spatiotemporal scales. This commentary critically analyzes these transformative findings, discussing how they resolve long-standing questions, diverge from established models, and open new paths for understanding and treating neurological diseases.

## 2. Novel Roles of Astrocyte Leaflets

**Figure 1:**
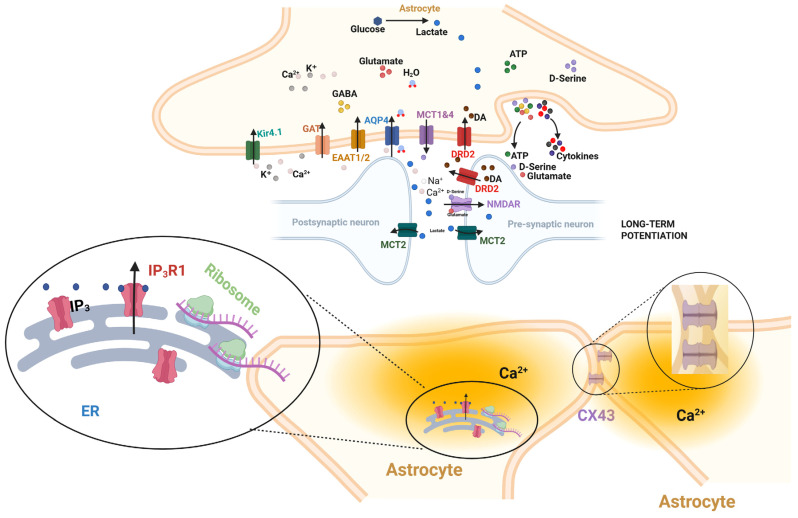
The Leaflet as a Specialized Astrocyte Computational Domain (created with biorender.com; BioRender, Toronto, ON, Canada).

The term “leaflet” has existed in the electron microscopy literature for decades, referring to the thinnest perisynaptic astrocytic processes that were historically viewed as passive, structural extensions, which were mere anatomical sheaths that provided physical isolation and supported basic homeostatic functions like neurotransmitter uptake and ion buffering. In this traditional context, their role was purely supportive, and their functional capacity remained speculative due to imaging limitations [2]. Benoit et al. (2025) fundamentally redefine this structure from a passive anatomical feature into a specialized functional domain [16]. Using vHREM and focused ion beam-scanning electron microscopy (FIB-SEM), they achieve nanoscale resolution that reveals leaflets not as uniform sheets, but as interconnected, gap-junction-coupled compartments containing unique ER saccules, distinct from perinuclear ER (Figure 1). This structural specialization alone suggests a dedicated locus for localized calcium signaling [16].

This schematic illustrates the tripartite synapse and the fundamental shift in understanding caused by Benoit et al. (2025) [16]. The central image shows the established roles of the astrocyte soma and major processes (e.g., glutamate recycling, general scaffolding). which also mediate metabolic and signaling crosstalk: Metabolic Support: Lactate is released from the astrocyte via MCT1/4 and taken up by the neuron (presynapse/postsynapse) via MCT2 to fuel neuronal activity; and signaling and receptors: Astrocytes release gliotransmitters like D-serine, which acts as a co-agonist alongside glutamate at postsynaptic NMDA receptors. The critical novelty is highlighted in the lower inset, which has been visually emphasized to reflect the findings of Benoit et al. [16]. This depiction shows the leaflet is not a passive sheath, but a complex micro-domain containing its own specialized endoplasmic reticulum (ER) saccules (purple), which are enriched with IP3R1 receptors to mediate fast, local calcium dynamics. These domains are functionally coupled to adjacent leaflets via gap junctions (CX43), enabling high-resolution processing of local synaptic input, distinct from the global, IP3R2-mediated signals of the central soma (main panel).

Methodologically, the study represents a significant leap, though it invites critical consideration. The vHREM/FIB-SEM data provide unprecedented 3D ultrastructural detail, but as fixed-tissue techniques, they cannot capture dynamic processes. The functional imaging using two-photon microscopy with GCaMP6f reveals highly localized calcium signals within leaflets, yet penetration depth and temporal resolution remain limiting. Moreover, the use of an astrocyte-specific triple *IP_3_R* knockout (*IP_3_R1,2,3*) to isolate leaflet-specific signaling, while decisive, may introduce compensatory adaptations that could affect physiological interpretation. Future acute, leaflet-specific manipulations will be essential to validate these findings.

The most transformative insight is the identification of IP_3_R1, not the somatically dominant IP_3_R2, as the key calcium-release channel in leaflets. This directly contradicts the long-standing paradigm that IP_3_R2 dominates astrocytic calcium signaling, a view largely built on observations of global somatic waves in *IP_3_R2*-knockout models. Benoit et al. resolve this discrepancy by revealing a critical subcellular specialization: IP_3_R2 governs slower, global somatic events, while IP_3_R1 drives rapid, localized signaling within leaflets [16]. This explains earlier reports of residual calcium activity in *IP_3_R2*-deficient mice and compels a re-evaluation of data from those models.

The discovery that leaflets form gap-junction-linked functional networks aligns with the classic concept of an astrocyte syncytium, but refines it by showing that integration occurs at the level of specialized leaflet domains rather than the whole cell. This modular organization supports earlier hypotheses that astrocytes can process multi-synaptic inputs, but now provides an ultrastructural and molecular basis for such computation. Conversely, it challenges the view of astrocytes as uniform integrators, proposing instead that leaflets enable parallel, sub-cellular computation with high spatiotemporal precision.

In summary, Benoit et al. shift the leaflet from a passive, structural element to an active, signaling-competent integrator [16]. The “old” role was passive ensheathment and homeostasis; the “new” role is active synaptic integration, coincidence detection, and circuit-level modulation. This redefinition not only resolves long-standing questions about how astrocytes integrate multi-synaptic information but also opens new avenues for investigating brain pathophysiology and therapy.

## 3. Physiological Significance

Physiologically, astrocyte leaflet domains enable astrocytes to process and modulate neuronal synaptic ensembles. By integrating calcium from clustered via leaflets, the astrocyte, leaflets could coordinate gliotransmitter release, influencing local neuronal circuits. For instance, domain-wide signals might synchronize vascular responses, coupling metabolism to activity [17]. In learning, leaflet integration could facilitate metaplasticity, where astrocyte-derived D-serine enhances NMDA-dependent LTP across related synapses [18]. Gap junctions allow electrotonic coupling, potentially propagating signals across astrocyte networks, as seen in hippocampal sharp-wave ripples [19]. ER saccules suggest localized calcium release, enabling leaflet autonomy from somatic control, thus supporting parallel processing. In development, leaflets may guide synaptogenesis; in homeostasis, they buffer ions and maintain synaptic fidelity. This “leaflet code” adds a glial layer to neural computation, where astrocytes act as integrators of multi-synaptic inputs, enhancing brain efficiency.

## 4. Pathological Implications

Pathologically, leaflet dysfunction could be implicated in human neurological diseases (NDs). In AD, the pathological atrophy and retraction of fine astrocytic leaflets are an early hallmark of synaptic dysfunction [20]. This loss of synaptic coverage directly compromises the leaflet’s ability to utilize its IP_3_R1-driven localized Ca^2+^ dynamics to maintain clearance mechanisms (such as efficient Aβ endocytosis and degradation) and volume homeostasis, which exacerbates toxicity.

In conditions characterized by rapid, uncontrolled signaling, such as epilepsy or ischemia, the gap-junction coupling (CX43) between leaflet domains could become maladaptive. This enhanced coupling can transform localized activity into a pathological syncytium, promoting hypersynchrony and wave propagation across neural circuits [21].

## 5. Developing Therapeutic Strategies

Therapeutically, targeting leaflet domains offers novel strategies. Specifically, the identification of IP3R1 as the driver of leaflet calcium dynamics offers a new mechanistic target for Aβ pathology. While direct experimental evidence linking IP3R1-specific modulation to Aβ clearance is still emerging, it is well-established that astrocytic structural processes are the primary sites for Aβ endocytosis and degradation [15,22]. Consequently, we hypothesize that IP3R1 agonists could enhance the functional integration of these leaflet domains, thereby restoring the calcium-dependent motility and surface area engagement required for efficient Aβ clearance, a process often impaired in Alzheimer’s disease [23].

However, clinical translation faces significant hurdles. As noted by critics of current pharmacological approaches, systemic administration of general IP_3_R1 agonists or non-selective gap junction blockers (e.g., carbenoxolone) lacks the necessary precision and would cause detrimental off-target effects across the CNS and periphery. Furthermore, while viral vectors (e.g., AAV) achieve cell-type specificity, current technology lacks the necessary subcellular targeting capability to deliver cargo specifically to the peripheral leaflet domains. Future therapeutic development must therefore prioritize solving these engineering challenges: identifying unique protein signatures of leaflets to enable the design of modified AAVs or smart compounds that modulate IP_3_R1-dependent synaptic signaling without disrupting the global, IP_3_R2-mediated functions of the astrocyte soma.

## 6. Challenges and Future Perspectives

Despite promises, multiple challenges exist. Imaging leaflets in vivo require super-resolution techniques, like stimulated emission depletion (STED)/expansion microscopy, limited by tissue penetration. Functional manipulation is hindered by astrocyte heterogeneity. In addition, leaflet-specific drivers are scarce, complicating optogenetic/chemogenetic targeting. Currently pathological modeling is constrained. The fly or mouse leaflets may not mirror primate complexity. The translation from animal model studies to humans faces species differences. The present AD mice models (e.g., 5xFAD) underexplore leaflet changes, necessitating investigation in human models, such as two-dimensional (2D) and three-dimensional (3D) induced pluripotent stem cells (iPSC)-astrocyte co-culture models. Furthermore, ethical concerns arise with glial editing (e.g., off-target neuronal effects), and clinical trials must address variability in leaflet responses in human neurological disorders.

However, future research could leverage artificial intelligence (AI)-driven modeling to simulate leaflet integration, predicting circuit outcomes. Advanced optogenetics (e.g., leaflet-targeted opsins) could dissect domain functions in behaving animals. Multi-omics (spatial transcriptomics, proteomics) studies could map leaflet-enriched molecules, identifying druggable targets. Human patient stem cell-derived organoid models with vascularized leaflets could be used to bridge preclinical gaps. Therapeutically, leaflet-inspired nanomaterials might mimic domain signaling for synapse repair. Furthermore, integrating leaflet biomarkers with blood-based assays (e.g., GFAP) could enable early detection of human NDs. Ultimately, the breakingthrough achievements from Benoit et al.’s work heralds a glial-centric era, where astrocytes are therapeutic protagonists, transforming management and intervention strategies for human neurological disorders [16]. 

## 7. Conclusions

Building on decades of glial research, the novel findings on astrocyte leaflets redefine astrocytes as synaptic integrators via leaflet domains. Physiologically, the identification of roles of astrocyte leaflets uncovers multi-scale computation within neuronal circuits. Pathologically, pathological factors to disrupt astrocyte leaflets may fuel NDs. Although therapeutic strategies targeting astrocyte leaflets hold promise, challenges in imaging and specificity must be overcome. Future investigation on astrocyte leaflets will unlock astrocyte potential and revolutionize brain disease treatments.

## Data Availability

No new data were created or analyzed in this study.

## Abbreviations

AQP4: Aquaporin-4; ATP: adenosine triphosphate; Ca^2+^: calcium ion; CX43: connexin-43; DA: dopamine; DRD2: dopamine receptor D2; EAAT1/2: excitatory amino acid transporter 1/2 (GLAST/GLT-1); ER: endoplasmic reticulum; GAT: GABA transporter; GABA: gamma-aminobutyric acid; IP_3_: inositol 1,4,5-trisphosphate; IP_3_R1: inositol 1,4,5-trisphosphate receptor type 1; Kir4.1: inwardly rectifying potassium channel 4.1; K^+^: potassium ion; MCT1/4: monocarboxylate transporter 1/4; MCT2: monocarboxylate transporter 2; Na^+^: sodium ion; NMDAR: N-methyl-D-aspartate receptor.
